# Supplementary figures and images for: Factors affecting the relative abundance in an overfished stock: red grouper (Epinephelus morio) in the Southeastern Gulf of Mexico
Source: PeerJ. 2023 Nov 20;11:e16490. doi: 10.7717/peerj.16490 (PMC10666610; doi:10.7717/peerj.16490)

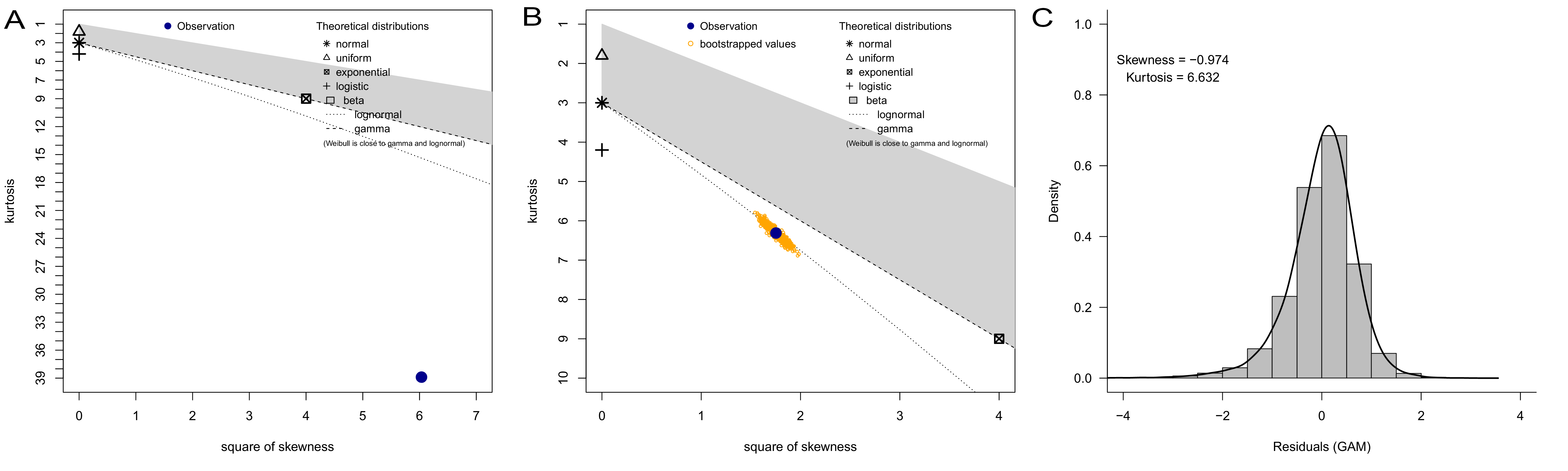

Supplement: Supplemental Information 1 — A) Cullen and Frey graph of catch-per-unit-effort behavior (blue point) B) Cullen and Frey graph of log-normal transformed catch-per-unit-effort behavior (blue point) with 1,000 bootstrap (gold points) C) Histogram of residuals of the best generalized additive model and density curve (black solid line). [file peerj-11-16490-s001.png]

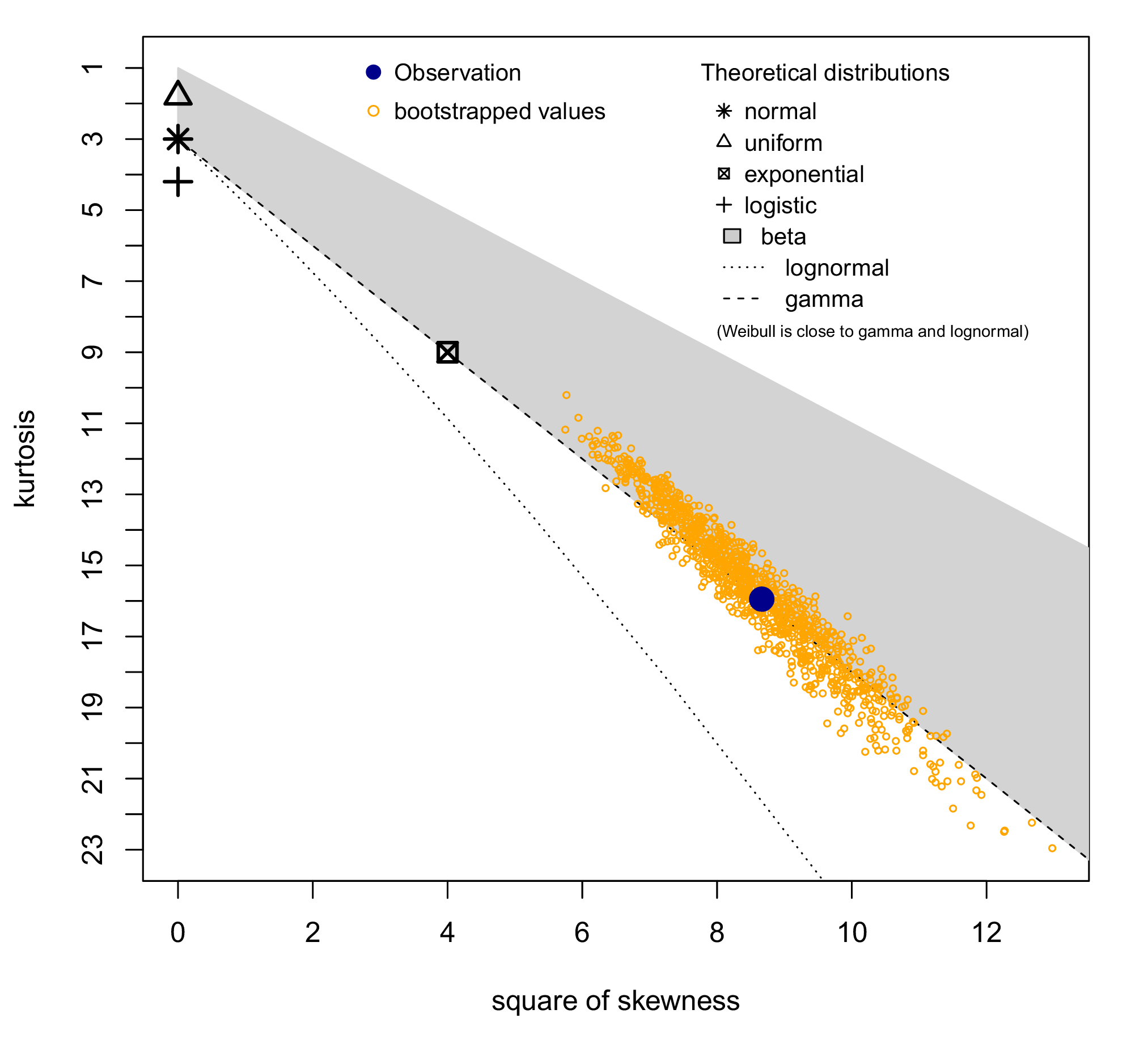

Supplement: Supplemental Information 2 [file peerj-11-16490-s002.png]
